# A reinforcement learning enhanced pseudo-inverse approach to self-collision avoidance of redundant robots

**DOI:** 10.3389/fnbot.2024.1375309

**Published:** 2024-03-28

**Authors:** Tinghe Hong, Weibing Li, Kai Huang

**Affiliations:** School of Computer Science and Engineering, Sun Yat-sen University, Guangzhou, China

**Keywords:** reinforcement learning, inverse kinematics, redundant robots, self-collision avoidance, sim to real

## Abstract

**Introduction:**

Redundant robots offer greater flexibility compared to non-redundant ones but are susceptible to increased collision risks when the end-effector approaches the robot's own links. Redundant degrees of freedom (DoFs) present an opportunity for collision avoidance; however, selecting an appropriate inverse kinematics (IK) solution remains challenging due to the infinite possible solutions.

**Methods:**

This study proposes a reinforcement learning (RL) enhanced pseudo-inverse approach to address self-collision avoidance in redundant robots. The RL agent is integrated into the redundancy resolution process of a pseudo-inverse method to determine a suitable IK solution for avoiding self-collisions during task execution. Additionally, an improved replay buffer is implemented to enhance the performance of the RL algorithm.

**Results:**

Simulations and experiments validate the effectiveness of the proposed method in reducing the risk of self-collision in redundant robots.

**Conclusion:**

The RL enhanced pseudo-inverse approach presented in this study demonstrates promising results in mitigating self-collision risks in redundant robots, highlighting its potential for enhancing safety and performance in robotic systems.

## 1 Introduction

Kinematically redundant robots possess more degrees of freedom (DoFs) than required to perform a user-specified task. Therefore, redundant robots deliver more advantages in human-robot interactions. However, greater flexibility originated from redundant DoFs increases the risk of self-collision, especially when the human operator forces the end-effector to move close to the robot's own links. Moreover, the robot links are constantly moving, which makes self-collision avoidance more difficult.

The crux in the self-collision problem of redundant robots lies in the difficulty of determining appropriate inverse kinematics (IK) (Paul and Shimano, 1978) solution. For non-redundant robotic arms, providing a specific position and orientation for the end effector typically results in no more than 32 IK solutions (Tsai and Morgan, 1985). However, in the case of redundant robotic arms, there often correspond an infinite number of solutions. This makes it challenging to determine an appropriate IK solution to avoid self-collision.

Firstly, it is difficult to obtain the closed form solution of redundant robots directly (Zaplana and Basanez, 2018) and it is also burdensome to meet the constraints of self-collision avoidance. Secondly, the IK solution should be consistent with the mechanical properties of the robot arm, i.e., the joint variables need to be bounded to ensure the smoothness of the motion. Finally, the solution should have some generality to migrate on multiple robots.

There are some limitations in the existing IK solutions. Some researchers have proposed numerical methods for solving inverse kinematics problems, such as the Jacobian matrix inversion-based solution (Colomé and Torras, 2015), the transposed Jacobian matrix-based solution (Wolovich and Elliott, 1984), and the damped least squares (DLS) solution (Nakamura and Hanafusa, 1986). Based on the current joints' status and the Denavit-Hartenberg (D-H) parameters (Denavit and Hartenberg, 1955) of a given robot, the end-effector can be continuously controlled under the guidance of one predetermined path. Unfortunately, numerical methods only provide a feasible set of IK solutions, which cannot be selected in complex cases. In contrast, Heuristic-based methods transform the IK problem into an optimization problem and select adaptive solutions. Momani et. al used a genetic algorithm to solve the IK problem and obtains a continuous smooth solution (Momani et al., 2016). In Rokbani and Alimi (2013) and Dereli and Köker (2018), different variants of the particle swarm optimization were presented to solve the IK problem for redundant robots. In Dereli and Köker (2020), a quantum particle swarm was proposed to compute the IK solutions of a 7-DoF robot. Koeker and Cakar improve the control accuracy of a robotic arm by combining neural networks, simulated annealing, and genetic algorithms (Köker and Çakar, 2016). Although heuristic methods can address IK problems under constraints, the dynamic nature of the robotic arm's motion creates a constantly changing environment, making it challenging for heuristic methods to converge. Furthermore, heuristic approaches often focus on solving for a specific state of the environment, overlooking the temporal and spatial continuity of the robotic arm's movement.

In contrast to traditional methods, machine learning approaches offer greater adaptability to complex tasks in controlling robots. In Cao et al. (2022, 2023a,b), authors have employed deep learning techniques to control robotic arms for grasping tasks. In a variety of machine learning approaches, Reinforcement learning (RL) (Sutton and Barto, 2018) involves a method that an agent explores the environment to achieve a maximum reward. Therefore, RL is suitable for finding an appropriate IK solution for a redundant robot under the constraint of collision avoidance. In Bing et al. (2023a,b), the authors employed meta-RL to control robots in simulated environments to achieve specific objectives. In Bing et al. (2022b), the authors utilized RL to control the locomotion of a snake-like robot. However, in self-collision avoidance situations, there are challenges in sample acquisition. Direct control of robot joints by reinforcement learning agents may cause difficulties in obtaining successful samples. In contrast, when controlling robot joints using the traditional IK method, collision samples are rare, which makes it difficult for the agent to learn how to avoid collisions.

To address the issues mentioned above an RL-enhanced pseudo-inverse approach is proposed in this paper. The RL solver does not directly control the robot, but imposes an interference to the pseudo-inverse solver to avoid self-collision of the robot. The main contributions of this paper are listed as follows:

- Firstly, an RL-enhanced pseudo-inverse solution method is proposed. In this approach, the RL agent outputs interference. The pseudo-inverse solver incorporates these interference into the computation to obtain an IK solution for robotic positioning with self-collision avoidance.
- A novel replay buffer is designed to adjust sample proportions under self-collision avoidance scenarios. This enhances the learning efficiency of the agent by elevating the diversity of samples in the buffer.
- Finally, a simulated training and testing environment was established in CoppeliaSim, and the effectiveness of the proposed method is validated through simulations and experiments using the Frank Emika Panda robotic arm.

## 2 Related work

Collision avoidance is always one of the critical issues to be solved in robotic arm control. The method in Guo and Hsia (1990) and Cheng et al. (1998) maximizes the distance between the robot arm and the obstacle to avoid collisions, but it is unnecessary to always maximize the distance when the robot is far away from the obstacle. In Duguleana et al. (2012), the authors propose an improved quadratic programming(QP) problem formulation, representation of the collision-free scheme as a dynamically updated inequality constraint. Haviland and Corke (2021) present a motion controller which is wrapped into QP. The controller can avoid static and dynamic obstacles while moving to the desired end-effector pose.

As an intelligent learning method, RL does not require an accurate model or prior knowledge, thus providing a new solution to the complex redundant robot control problem. The authors of Al-Hafez and Steil (2021) take the concept of redundancy resolution and propose a policy search with redundant action bias to control the motion of the robotic arm and avoid collisions by maximizing the distance between the linkage and the obstacle. In Li et al. (2022) the authors propose a framework that employs deep reinforcement learning (DRL) to find the most efficient path in Cartesian space and to compute the most energy-efficient solution for robot IK. In Bing et al. (2022a, 2023c), the authors trained robotic arms to evade obstacles and grasp targets using a method based on Hindsight Goal Generation.

In Martin and Millán (1997) the authors employ proximity sensors and reinforcement learning methods to solve the self-collision problem of redundant robotic arms. However, this approach is specific to two-dimensional robotic arms and difficult to extend to three-dimensional space. In Agarwal et al. (2016) and Schappler and Ortmaier (2021), the authors employed the null space projection to address singularity avoidance in three-axis planar robots and six-axis serial robots, respectively. In comparison to singularity avoidance, self-collision avoidance requires more consideration of the robotic arm's structure, as arms with different structures have varying joint positions during motion, making it more challenging to predict the location of the links.

In summary, the issue of self-collision in robotic arms becomes increasingly significant with the growth of joint complexity, and there is currently limited attention given to this problem. Traditional collision avoidance methods typically focus on obstacles with fixed or uniform motion or impose restrictions on the number of joint angles of the robotic arm. The proposed method in this paper aims to avoid irregularly moving robotic arm links and is insensitive to both the number and structure of joints in redundant robotic arms.

## 3 Mathematical model

Generally, the control of a robotic arm is associated with a mapping from the work space to the joint space. However, it is difficult to directly calculate the relationship between the change in end-effector's pose and the change in joints' states. Therefore, a common approach is to map the change in pose to the change of joint velocity, and the work space and joint space are related by a Jacobian matrix in the mapping.

Set the desired velocity of the end-effector of the robot arm to be *ẋ*, which is a 6-dimensional vector (three translations and three rotations), and let *J* denote the Jacobian matrix. The joint velocity of the robot arm is an *n*-dimensional vector, referred to as $\dot{q}$, where *n* represents the number of DoFs of the robot. The velocity of the end-effector *ẋ* could be obtained from $\dot{q}$ and *J* in Equation (1):


(1) $$\begin{array}{l} ẋ=J\dot{q}. \end{array}$$

Then, based on the pseudo-inverse method, it yields Equation (2):


(2) $$\begin{array}{l} \begin{array}{c} \min∥\dot{q}{∥}^{2}\\ \text{subject to }ẋ=J\dot{q}. \end{array} \end{array}$$

The pseudo-inverse method uses the minimum joint velocity as the optimization objective to improve the motion efficiency. However, this optimization objective cannot satisfy the need for self-collision avoidance. As a result, a controllable interference $\dot{i}$ is added, where $\dot{i}$ represents a vector with the same dimension as $\dot{q}$ in Equation (3):


(3) $$\begin{array}{l} \begin{array}{c} \min∥\dot{q}+\dot{i}{∥}^{2}\\ \text{subject to }ẋ=J\dot{q}. \end{array} \end{array}$$

According to the Lagrange multiplier method (Boyd and Vandenberghe, 2004), it leads to


(4) $$\begin{array}{l} \dot{q}=J^{T}{\left(JJ^{T}\right)}^{-1}\left(ẋ+J\dot{i}\right)-\dot{i}. \end{array}$$

Define *J*^†^ = *J*^*T*^(*JJ*^*T*^)^−1^. Since *JJ*^†^ = *I*,*J*^†^ is the right pseudo-inverse of the Jacobian matrix *J*. Therefore, Equation (4) is equivalent to


(5) $$\begin{array}{l} \dot{q}=J^{†}\left(ẋ+J\dot{i}\right)-\dot{i}. \end{array}$$

The right pseudo-inverse of the Jacobian matrix can be solved with any of the optimization solvers without affecting the computation of $\dot{q}$. The existence of the pseudo-inverse solution with interference is not affected since the inverse matrix is replaced by the pseudo-inverse and *J*^†^ necessarily exists.

Lemma 1. After adding the interference, the resulting $\dot{q}$ still allows the end-effector to move with the desired velocity *ẋ*.

*Proof*. Multiplying *J* left on both sides of Equation (5), then get Equation (6):


(6) $$\begin{array}{l} \begin{array}{c} J\dot{q}=JJ^{†}\left(ẋ+J\dot{i}\right)-J\dot{i}\\ =ẋ+J\dot{i}-J\dot{i}\\ =ẋ. \end{array} \end{array}$$

This proves that $\dot{q}$ with interference $\dot{i}$ can generate the desired velocity for robot motion control.

## 4 Proposed RL enhanced controller

**Figure 1:**
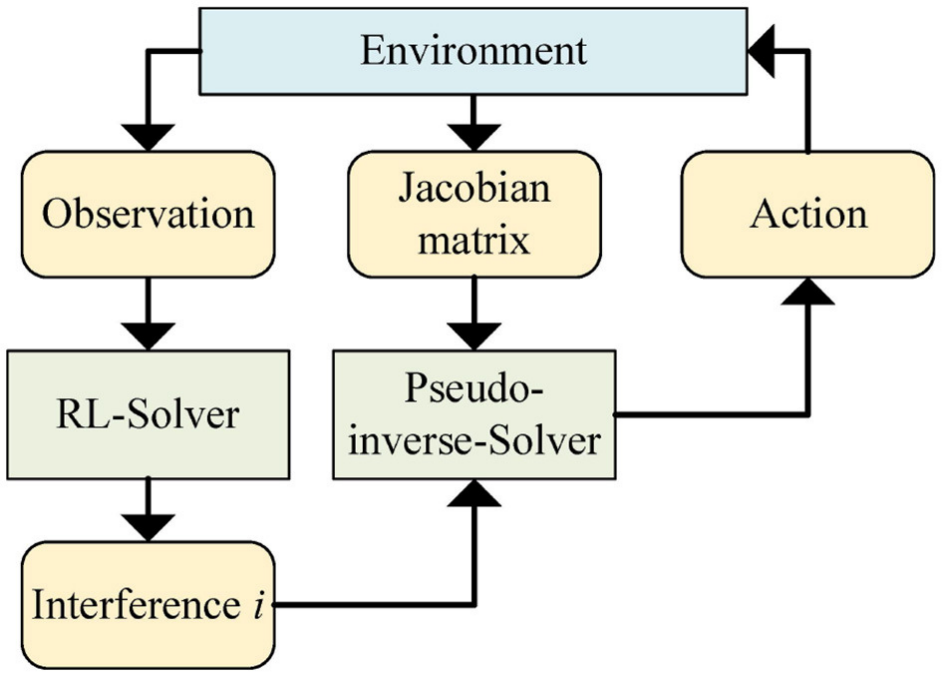
Structure of RL enhanced pseudo-inverse controller.

The proposed controller contains two solvers, the RL solver and the pseudo-inverse solver. The RL solver accepts observation from the environment and returns interference $\dot{i}$. The pseudo-inverse solver accepts the Jacobian matrix *J* from the environment and calculates the velocity $\dot{q}$ of robot joints in the current state by combining interference $\dot{i}$ according to the method in Equation (5). A series of actions will be obtained through the controller. The structure diagram of the controller is shown in Figure 1. The pseudo-inverse solver has been explained in Section 3. Next, we will present the key elements of the RL solver and the RL network structure.

### 4.1 Observation space

The RL agent receives the environment information through the observation space at each step. In RL, the correct choice of observation space parameters is crucial as the agent needs the correct set of information to understand the causality of a given reward based on the behavior.

The observation space *o*^*t*^ is given in Table 1. ϕ is an n-dimensional vector to present the current robot joints' angle. Define *ṗ* as the difference of coordinates between the target point and the current robot end-effector. The target point position coordinates is denoted as *ṗ*_*tar*_, and the end-effector position is denoted as *ṗ*_*ee*_. *ṗ*, *ṗ*_*tar*_, and *ṗ*_*ee*_, are 3-dimensional vectors. The 3 dimensions correspond to the coordinates of the Cartesian coordinate system in the *x*, *y* and *z* directions. *ṗ* can be obtained from


(7) $$\begin{array}{l} ṗ={ṗ}_{tar}-{ṗ}_{ee}. \end{array}$$

**Table 1:** The observation space *o*^*t*^ of the RL controller.

**Symbols**	**Description**
ϕ	Current joints angle
*ṗ*	Difference between target and current position
*ṙ*	Difference between initial and current rotation

Define *ṙ* as the angle difference between the end-effector at the beginning and the current position. The initial end-effector rotation is denoted as *ṙ*_*init*_, and the current rotation is denoted as *ṙ*_*cur*_, then obtain the rotation difference from Equation (8):


(8) $$\begin{array}{l} ṙ={ṙ}_{init}-{ṙ}_{cur}. \end{array}$$

Similarly, *ṙ*, *ṙ*_*init*_, and *ṙ*_*cur*_ are all 3-dimensional vectors that represent the rotation about the x, y, z axes in the Cartesian coordinate system.

In summary, an overall (*n*+6)-DoFs observation space is used in this work. In the simulation of this paper, the position and rotation information of the end-effector are obtained from the simulator directly. For the robotic arm in a physical environment, this information can be estimated using forward kinematics.

### 4.2 Action space

At time *t*, the final output of the controller is an *n*-dimensional vector *a*^*t*^, denoted as $\dot{q}$ in the Equation (5), where *n* represents the DoF of the robotic arm. The values of *a*^*t*^ fall within the range of [-1, 1] and are transformed linearly to correspond to the velocities of the respective joints. The RL solver produces the interference vector *i*^*t*^, which is also an *n*-dimensional vector, representing the interference $\dot{i}$ in the Equation (5) at time *t*. It is worth noting that this paper primarily focuses on the robot's kinematics, and joint torques are beyond the scope of this study.

### 4.3 Single-step reward

When the RL solver introduces excessive disturbance to the Pseudo-inverse solver, it can result in deviations of the end effector's motion from the target trajectory or unexpected rotations. For instance, when the joint motion of the robotic arm exceeds limits, it may cause a significant deviation of the end effector from the designated motion trajectory.

To prevent the occurrence of the aforementioned phenomena, it is essential to calculate rewards for each step of the RL solver's output. The reward function for the single-step reward comprises translation and rotation components.

For the translation component, we take the current velocity vector of the end-effector, the angle θ with the target direction vector, and compute cos(θ). In the following steps, we take the single-step translation reward as cos(θ)−1 to ensure it is negative. When the end-effector moves exactly in the specified direction, the reward is set as the maximum value of 0. The translation component is denoted as *R*_*l*_, it could be found that,


(9) $$\begin{array}{l} R_{l}=\frac{ṗ\cdot v_{ee}}{\left|ṗ||v_{ee}\right|}-1, \end{array}$$

where *v*_*ee*_ is a 3-dimensional vector to represent the current translation velocity of the end-effector.

For the rotation component, as this paper primarily focuses on position inverse kinematics issues, we take the default rotation of the end-effector to make a difference with the current rotation. The 2-norm is taken for the resulting vector. Finally, the obtained value is divided by a factor *k* to balance the value with the translation component. With the translation component, a non-positive value is taken for the obtained value, which is rewarded as a maximum value of 0, when the end-effector remains theinitial rotation. Let the rotation component be *R*_*r*_, it can be computed as follows,


(10) $$\begin{array}{l} R_{r}=-ṙ/k, \end{array}$$

where *ṙ* be computed from Equation (7). In this paper, *k* is set as 100. The coefficient *k* balances the translation reward and rotation reward, avoiding that one reward is too large and agent ignores the other one.

Combining Equations (9, 10), yields the reward function for each step


(11) $$\begin{array}{l} R_{s}=R_{l}+R_{r}. \end{array}$$

When the end effector moves in the vicinity of the robotic arm according to the specified trajectory, the single-step reward approaches zero. However, if the robotic arm's joints exceed limits or other geometric structural issues impede its normal motion, the single-step reward significantly decreases. This encourages the RL solver to avoid situations where the robotic arm becomes stuck.

It is noteworthy that the rewards returned by Equation (11) are for each step and do not encompass the rewards for each episode. Episode rewards will be explained in the next subsection.

### 4.4 Episode rewards

The single-step reward calculation involves the rewards received by the agent for each action taken. Episode rewards, on the other hand, are computed based on the outcomes after the completion of an episode. Since the position relationship between adjacent state-action pairs is lost during retraining after replays are placed into the buffer, it is necessary to finalize the reward allocation for each episode before adding the replay to the buffer. In this paper, a Monte Carlo-like method is employed, where the final reward accumulated at the end of an episode is propagated backward and assigned to all steps within that episode.

Assume there are *k* replays in one episode, meaning that this episode takes *k* steps. *R*_*j*_ is the *j*th step reward in episode buffer, then adjusted the rewards in episode buffer as


(12) $$\begin{array}{l} R_{a}=R_{j}+\gamma^{k-j}R_{end}, \end{array}$$

where *R*_*a*_ is the adjusted reward. *R*_*end*_ is the end reward of an episode, based on if the episode results in success or failure, its value is represented by Equation (13).


(13) $$R_{e}=\{\begin{array}{cc} R_{pos} & when\;reach\;the\;target\\ R_{neg} & other, \end{array}$$

where *R*_*pos*_>*R*_*neg*_, it indicates that the reward obtained upon successfully reaching the target position exceeds the reward obtained upon failure.

The intuition behind Equation (12) is to reinforce the correlation between adjacent replays. If an action results in the robotic arm moving toward a collision direction, the reward for the corresponding state-action pair is correspondingly reduced.

While it is possible to enhance training performance by magnifying the reward/punishment values at the conclusion of each episode and transmitting this value through the Bellman equation, it is observed that directly amplifying these values during training often led to frequent training failures. The approach outlined in Equation (12), however, facilitates more successful training. We hypothesize that the use of Equation (12) disperses the reward/punishment values across multiple records, preventing issues associated with excessively large gradients.

### 4.5 Dynamic balancing replay buffer

The role of the replay buffer in RL is to improve the utilization of samples and to enable the agent to train offline. The rewards generated by the interaction between the agent and the environment are stored in the replay buffer.

The usual approach is to add the rewards returned by the environment directly to the replay buffer, then randomly select some replays from the buffer to train the agent. However, there are two challenges in this paper.

One of the challenges is that the movement of the robot is continuous, and collisions occur not triggered by only one action. The usual approach destroys the cause-and-effect relationship between adjacent actions.

The other challenge is that during the training of the combined controller, the domination of any solver causes reward/penalty sparsity. For example, when the RL solver dominates, the untrained RL solver makes the robot always collide and causes it difficult to converge. On the other hand, when the pseudo-inverse solver dominates, the robot rarely collides, which leads the RL solver difficult to get trained because of the lack of collision samples.

The first challenge was addressed in the preceding subsection through an episode-based approach. As for the second challenge, the proposed solution in this paper involves the introduction of a dynamic balancing mechanism for the replay buffer.

Let *info* return at the end of an episode. Return *True* when the end-effector successfully reaches the target, otherwise return *False*.

Let the total number of steps from successful episodes in the current replay buffer to *n*_*s*_ and the total number of steps from failed episodes to *n*_*f*_.

The replays in episode buffer will be added to the replay buffer only when:

1. *info* = *True and n*_*s*_ ≤ *n*_*f*_, or
2. *info* = *False and n*_*s*_>*n*_*f*_

This dynamic balancing mechanism ensures that the number of successful and failed steps in the replay buffer is similar, thus avoiding the problem of difficult convergence due to the lack of samples.

### 4.6 Network and training

Given the input (observation *o*^*t*^) and the output (action *a*^*t*^), the details of the network structure are introduced. We train our agent using the TD3 (Fujimoto et al., 2018) based on the Actor-Critic architecture, thus requiring two Critic networks and one Actor network. Each Critic network contains two fully connected hidden layers that act as non-linear function approximators of Q value. The dimension of the input layer is the sum of the dimensions of *o*^*t*^ and *a*^*t*^. Both hidden layers have 128 PReLU (He et al., 2015) units. The last layer outputs a Q value. The Actor network also contains two fully connected hidden layers. The input layer has the same dimensions as *o*^*t*^. Both two hidden layers have 128 ReLU units, with the last layer outputting the interference $\dot{i}$.

Theoretically, any deterministic policy RL algorithm can be applied to our method, but the RL algorithm with a stochastic policy may cause the robot arm to jitter, such as the SAC (Haarnoja et al., 2018) we have tested which shown in Section 5.

## 5 Simulation and experiment

In this section, simulative and experimental validations were conducted to assess the effectiveness of the proposed approach in avoiding self-collision for redundant robotic arms. In both simulation and experimentation, the end effector's motion trajectory was defined to compel its movement in proximity to the robotic arm's own structure. The experimental scenarios simulated situations that might occur when an operator directly manipulates the end effector of the robotic arm.

Coppeliasim is a kind of mainstream robot simulator, which can simulate the motion and collision detection of robot arms. In the simulation, a built-in Franka Panda robotic arm with 7-DoFs are employed. The initial joint angles are set to [0°, −17°, 0°, −126°, 0°, 114°, 45°]. The initial coordinate position of the end-effector is [0.499*m*, 0*m*, 1.189*m*].

The goal of the simulation and experiment is to guide the end-effector motion to the target point. The target points are generated in a hemispherical space of 0.5*m* radius around the first joint of the robot arm. A series of path points are generated at 0.01 m intervals along a straight line between the initial position and the target point position to force the end-effector to move near the robot arm itself. When the distance between the end effector and the target is less than 0.001 meters, the end effector is considered to have reached its destination. Millimeter-level accuracy is deemed sufficiently precise given the range of motion of the robotic arm in this experiment. Additionally, the methods employed in this study are iterative, and demanding excessive precision would result in the control algorithm consuming an impractical number of steps during the final convergence process. In the simulation, the built-in collision detector in Coppeliasim is employed to detect whether the robotic arm has collided. PyRep (James et al., 2019) is employed to set up the reinforcement learning environment.

The platform configuration of training and simulation is listed as follows: CPU: i9-9900K; GPU: 2080ti; Memory: 64G. Coppeliasim version is 4.1 EDU and the operating system is Ubuntu 18.04 LTS.

### 5.1 Convergence validation simulation

**Figure 2:**
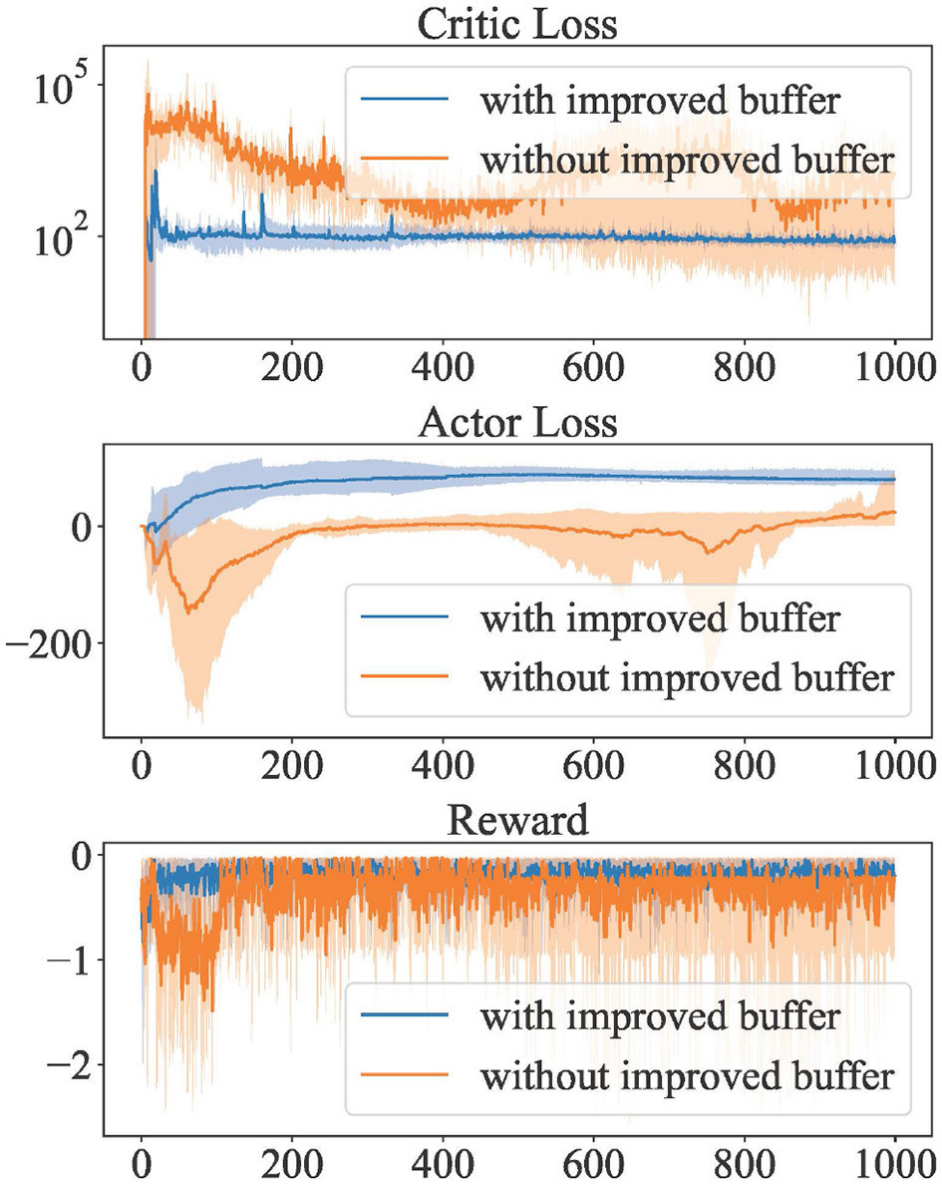
Convergence verification under initialization conditions dominated by RL solver.

This simulation compares the convergence with and without the use of our improved replay buffer. The algorithm has been trained for 1000 episodes, which is about 200,000 steps. The parameters employed during training are outlined in Table 2. For the sake of stability during training, Stochastic Gradient Descent (SGD) was utilized instead of momentum-based optimizers. The results shown in Figure 2 represent the upper and lower bounds and average values for 25 replicate simulations.

**Table 2:** Convergence verification parameter.

**Parameters**	**Value**
Episodes	1, 000
Critic learning rate	2 × 10^−4^
Actor learning rate	1 × 10^−4^

As can be seen from the Critic convergence curve, the improved replay buffer makes the algorithm converge more stably. On the other hand, the unimproved replay buffer affected Critic's convergence and even led to failure to converge. Since Critic estimates Q values of state-action pairs, it is verified that our improved replay buffer improves the accuracy of the agent's estimation of the environment.

While the lower Actor loss is better, the unimproved replay buffer leads to an inaccurate estimate of Critic. Thus the lower loss under the unimproved replay buffer is not convincing. In contrast, our improved replay buffer exhibits more stability compared to the unimproved replay buffer.

Benefiting from better stability, our improved replay buffer allows for higher rewards per step and lower volatility during multiple training sessions.

### 5.2 Single target positions arrival experiment

In this simulation, 1071 final target points are evenly distributed in the hemispherical space around the robot arm. In each episode, the end-effector of the robotic arm starts from the same initial position and follows a moving target to reach the final target point. The moving target moves in a straight line, forcing the end-effector to move close to the arm's own structure.

Six different methods are compared in this simulation. To compare methods without collision avoidance policies, PI (pseudo-inverse) and TJ (Jacobian Transpose) methods are introduced in this simulation as comparisons. NEO (Haviland and Corke, 2021) is introduced into the comparison as a non-learning-based obstacle avoidance method. The RL method uses only the TD3 algorithm as a comparison of not improving on the replay buffer. HER (Andrychowicz et al., 2017) is introduced as a method that improved the replay buffer in this comparison. Replay buffer in HER method uses future choosing strategy. It is difficult to predict the timing of collision occurrence in the simulation, thus the update of the replay buffer in HER method is to encourage the tracking of target points by the agent. OURS method is the enhanced pseudo-inverse method of reinforcement learning with an improved replay buffer proposed in this paper.

**Figure 3:**
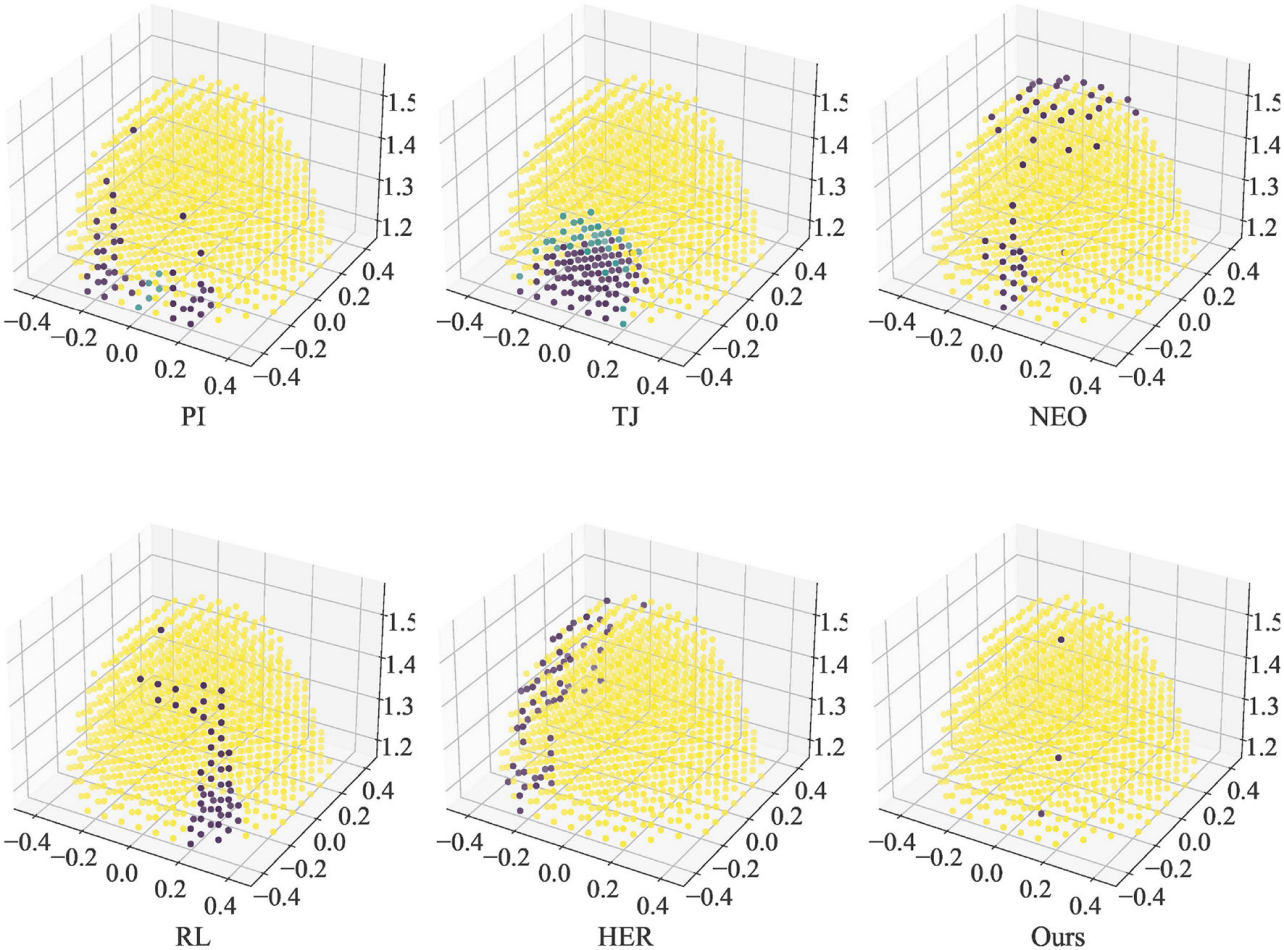
Ability of the end-effector to reach a single target position under control of different approaches. Yellow dots indicate successful arrival at the target. Green dots indicate that the number of steps is used up and the target is not reached (except NEO). Purple dots indicate collisions (except NEO).

The max number of steps for each episode is 1000. There are three outcomes for each episode, successful arrival at the target position (Success), exceeding the step limit but no collision (Run out), and Collision. The results of this simulation are summarized in Figure 3 and Table 3. The number of the three results in a single simulation is counted in the Success, Run out, and Collision columns respectively. Avg steps column counts the average number of steps spent in the episode of Success. The avg reward column counts the average reward per step. In this simulation, the reward is calculated according to Equation (11), reflecting the tracking performance of the end-effector on the target point under the control of different methods.

**Table 3:** Details of the end-effector reaching single position under the control of different methods.

**Methods**	**Success**	**Run out**	**Collision**	**Avg steps**	**Avg reward**
PI	1,025	5	41	97.68441	-0.04706
TJ	961	33	77	514.761	-0.18328
NEO	1,023	48	0	222.2605	-0.10953
RL	1,021	0	50	219.8609	-0.05474
HER	1,014	0	57	162.2745	-0.0493
OURS	1,068	0	3	234.6667	-0.06235

In Figure 3, yellow dots indicate the positions where the end-effector can reach the target successfully. Green dots indicate positions that cannot be reached before the steps run out. Purple dots indicate collisions when reaching these positions. The NEO method did not collide, so points indicating Run out are marked in purple for clarity in observation. The figure shows that moving the end-effector from the initial position to the back of the robot arm is challenging for the controller. The RL method without an improved replay buffer does not have an advantage over the traditional numerical method. The optimization-based collision avoidance approach NEO has difficulty in finding a suitable solution for avoiding irregularly moving robotic arm links, especially when a well-defined path is given.

According to the data in Table 3, it can be seen that our method has the highest arrival rate (99.72%), but the Avg reward is slightly lower. This could be attributed to a decrease in the end-effector movement speed under Agent control (resulting in a higher average number of steps compared to PI) and slight deviations from the planned trajectory, leading to a reduction in rewards. NEO is able to avoid collisions completely in this simulation but has more Run out cases. In the simulation, it was observed that NEO causes the robot arm to get stuck in certain poses and cannot continue tracking the target. Also, the NEO method prefers to find the trajectory freely, so it cannot track the target well under strict constraints on the trajectory, which is reflected by having a lower Avg reward. Attributed to encouraging target tracking, HER has lower Avg steps and higher Avg reward but does not do better in avoiding collisions and reaching the final target.

**Figure 4:**
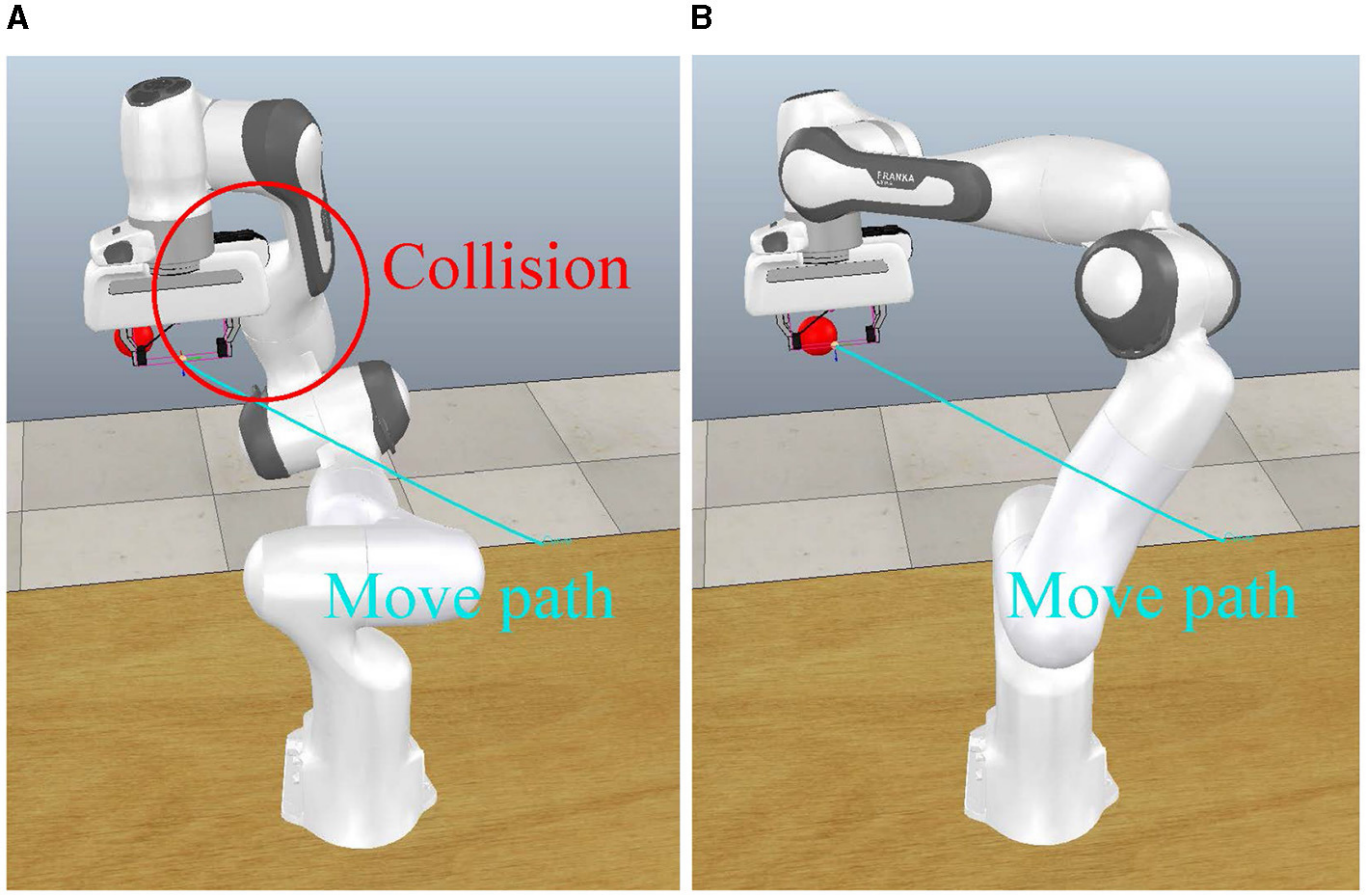
Path under different control methods. The cyan line depicts the motion trajectory of the end-effector, while the red sphere indicates the target position of the end-effector. Red circles highlight locations where collisions occur under the control of the pseudoinverse method. **(A)** PI posture. **(B)** Ours posture.

Figures 4A, B show how our method avoids self-collisions during tracing the target. The blue curve in the figure shows the trajectory of the end-effector. It can be seen that the end-effector maintains a smooth motion path under both methods. In Figure 4A, the PI method is employed to control the motion of the robotic arm. This resulted in a collision between the end-effector and the robot arm linkage at the red circle. In Figure 4B, our method controls the robot arm to rotate its own mechanism to move away from the motion path of the end-effector. Because of this behavior, our method can avoid self-collisions.

### 5.3 Three target positions arrival simulation

In each episode of this simulation, three final target positions are randomly generated. The controller needs to guide the end-effector to reach the three positions consecutively. Unlike the single target experiment, the initial of each segment of the moving robot arm cannot be predicted. The experiment is executed for 1,000 episodes. The upper limit of steps per episode is 3,000. The performance of the six methods is compared. The target positions are pre-generated to ensure that each method faces the same challenge.

The results of the simulation are shown in Table 4. Similar to Section 5.2, the controller guides the end-effector to reach the three final target positions in sequence and is recorded as Success. Failure to reach any target position or collision in the middle of the episode is recorded as run out or collision. The table also shows the average steps per episode and the average reward per step.

**Table 4:** Details of the end-effector reaching three different positions under the control of different methods.

**Methods**	**Success**	**Run out**	**Collision**	**Avg step**	**Avg reward**
PI	778	0	222	260.327	-0.08718
TJ	796	112	92	1294.823	-0.14426
NEO	742	160	98	528.74	-0.10586
RL	751	23	226	463.561	-0.08597
HER	723	5	272	393.234	-0.07653
Ours	931	0	69	504.456	-0.05887

The data in the Table 4 shows that our method has a higher successful arrival percentage than the method without the improved replay buffer, which indicates that the improved replay buffer enhances the performance of the RL algorithm. Our method also has an advantage over the traditional method, which indicates that the combined reinforcement learning and pseudo-inverse methods give the robot arm a better ability to avoid self-collision. The lower average reward for all methods compared to the single target position experiments is due to the longer average number of steps, thus generating more negative rewards.

### 5.4 Ablation experiments

The purpose of this section is to evaluate whether the improvements for different challenges improve performance. To this end, we repeat the experiments of the Section 5.2 section and keep all other parameter settings identical.

To verify the effectiveness of the reward adjustment in Equation (12), we select different γ for training and tested the training results in simulation. The results of the test are shown in Table 5.

**Table 5:** Details of simulated data with different success and failed buffer ratios.

**γ**	**Success**	**Run out**	**Collision**	**Avg step**	**Avg reward**
0.99	1,061	0	10	171.2754	-0.0412
0.95	1,041	0	30	164.7703	-0.05292
0.9	1,058	0	13	170.605	-0.04269
0.7	1,061	0	10	172.6872	-0.05023
0.5	1,050	1	20	168.5556	-0.04323
0.2	1,061	0	10	172.6872	-0.05024
0.1	1,042	0	29	166.549	-0.04163
0.05	1,053	0	18	169.4585	-0.04364
0.01	1,041	20	10	168.8571	-0.06217

As can be seen from the table, the adjusted reward has a positive effect on avoiding self-collision of the robot arm, but the effect does not increase linearly with γ. The success rate of tracking showed two peaks when γ is close to 0.2 and 0.7. In addition, the algorithm shows better stability when γ is set to 0.2 in repeated training.

To verify the effectiveness of the dynamic balancing mechanism, a set of simulations with different ratios of success and failed replay buffer are performed. The results of the simulation are shown in Table 6.

**Table 6:** Details of simulated data with different success and failed buffer ratios.

**Percent**	**Success**	**Run out**	**Collision**	**Avg step**	**Avg reward**
No balance	1,042	0	29	166.3119	-0.04251
50%	1,061	0	10	172.3688	-0.05038
75%	1,056	0	15	170.0355	-0.04462
100%	819	0	252	118.535	-0.04596

This simulation compares the tracking of end-effectors with four different ratios. In the No balance simulation, there is no limit on the percentage of successful and failed replay, and the percentage of failed replay is about 32.37% (135,480 of 418,525). The remaining simulations limit the percentage of failed replay to about 50%, about 75%, and 100%.

As can be seen in Table 6, Balancing successful and failed replay by 50%-50% can effectively reduce the probability of self-collision of the robot arm, although this increases the average number of steps and slightly reduces the average reward. Slightly more failed replay (75%) also reduces the likelihood of self-collision, but not as effectively as keeping it at 50%. The probability of collision increases when using failed replays entirely, due to the lack of successful samples resulting in the agent's inability to properly evaluate the environment.

Two conclusions can be drawn from the simulation results as follows. A) The dynamic balance replay proposed in this paper is effective in avoiding self-collision of the robotic arm. B) Appropriate discarding of some replay may have a positive impact on the behavioral strategy of the agent.

To examine the impact of distinct weights for *R*_*l*_ and *R*_*r*_ on the agent as stipulated in Equation (11), the reward function is configured as Equation (14)


(14) $$\begin{array}{l} R_{s}=\alpha R_{l}+\left(2-\alpha \right)R_{r}. \end{array}$$

The performance of the proposed apporach is evaluated under varying α values, and the results are presented in Table 7.

**Table 7:** Details of simulated data with different success and failed buffer ratios.

**Ratio α**	**Success**	**Run out**	**Collision**	**Avg step**	**Avg reward**
0.25	1,029	1	41	173.6502	-0.05646
0.5	1,038	0	33	174.3946	-0.05828
1	1,056	0	15	173.4608	-0.05111
1.5	1,032	0	39	172.9067	-0.05961
1.75	1,027	0	44	172.9543	-0.05935

From Table 7, it can be observed that the proposed method performs favorably when α is set to 1. Therefore, in this paper, α is chosen to be 1, as indicated in Equation (11).

### 5.5 Motion smoothness demonstration

To verify that the method in this paper not only smooths the end-effector motion but also keeps the velocity of the robotic arm joints smooth. All joint velocities for 200 consecutive steps were collected to verify the smoothness of the robot arm joint motion.

Let the Action of step *t* be ${\dot{q}}^{t}$. The acceleration ȧ^*t*^ is calculated by approximating the velocity of two adjacent steps as Equation (15)


(15) $$\begin{array}{l} {ȧ}^{t}={\dot{q}}^{t+1}-{\dot{q}}^{t}. \end{array}$$

To represent the acceleration as a single value, a first order norm for ȧ^*t*^ has been taken. After that, divide it by the number of joints to get the average acceleration ${\bar{a}}^{t}$ of the joints at step *t*. The formula is expressed as Equation (16)


(16) $$\begin{array}{l} {\bar{a}}^{t}=|{ȧ}^{t}|/n. \end{array}$$

**Figure 5:**
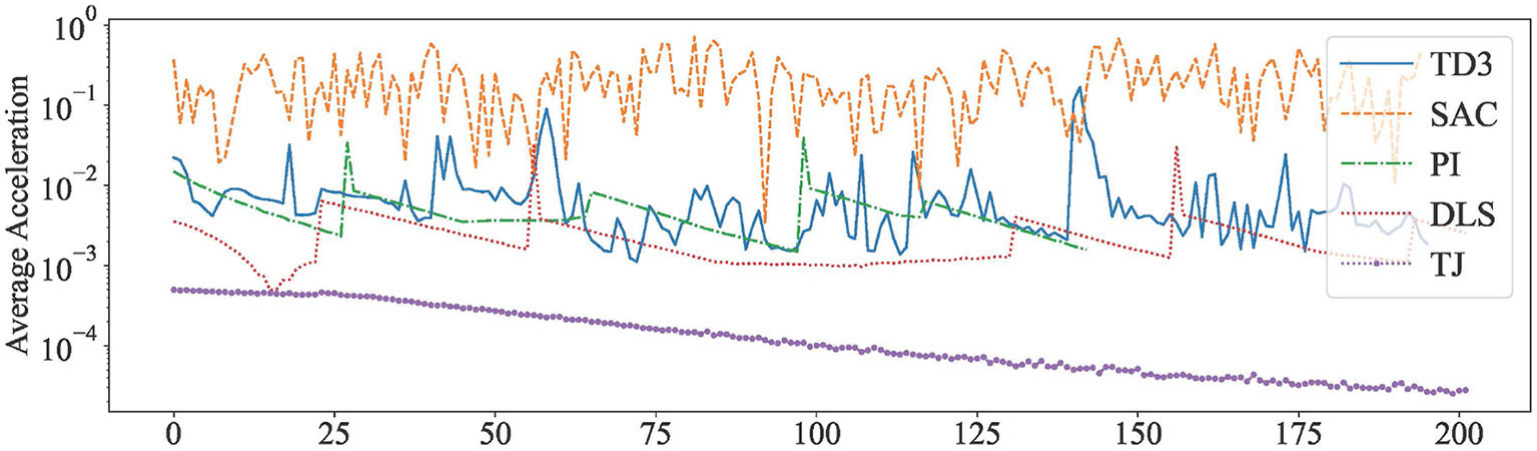
Average acceleration of robot arm joints under different methods of control.

To visualize the results, the acceleration curves of Our method (Combined deterministic algorithm TD3 and pseudo-inverse) and SAC (Combined stochastic algorithm and pseudo-inverse) have been plotted. As a comparison, the acceleration curves of PI, DLS, and TJ are also plotted. The results are shown in Figure 5, and detailed data are shown in Table 8.

**Table 8:** The average acceleration of each joint of the robot arm under different methods.

**Methods**	**Average**	**Maximum**	**Variances**
Ours	8.8e-3	0.17	2.8e-4
SAC	0.22	0.71	2.3e-2
PI	5.6e-3	0.05	3.7e-5
DLS	2.4e-3	0.03	9.9e-6
TJ	1.7e-4	5e-4	2.3e-8

As can be seen in Figure 5, the curves of both our method and the SAC method produce significant fluctuations. This indicates that the control of the reinforcement learning method caused the jitter of the robot arm joints. However, the acceleration range of our method is closer to that of PI, which is two orders of magnitude lower than that of the SAC method. This is because our controller employed a deterministic reinforcement learning algorithm and calculate the final result by numerical methods. The PI, DLS, and TJ methods have smoother acceleration profiles because they are calculated in a purely mathematical way, with large fluctuations only in the case of target changes.

In Table 8, the mean, maximum, and variance of the acceleration for each of the five methods in 200 steps are calculated. These data are used to reflect the variation of acceleration for the above methods.

Our method is closer to the numerical methods PI and DLS in terms of average acceleration and maximum acceleration as shown in Table 8. The average acceleration of our method is 3 orders of magnitude lower, while the variance of acceleration is 2 orders of magnitude lower than the SAC method. Therefore, when the robot arm is controlled by our method, not only the trajectory of the end-effector is smooth, but also the motion of the other joints of the robot arm is steady.

The TJ method has the best acceleration performance. However, as it is known from previous experiments, the TJ method requires more steps to reach the target, so its lower acceleration is due to its slow movement speed.

### 5.6 Experiment

In this experiment, our method is deployed on a real Franka Emika Panda robot. The goal of the experiment is to move the end-effector along a straight line to the rear of the robotic arm to examine the ability of the algorithm to avoid collisions. The initial position of the end-effector is [0.5, 0.0, 0.36], and the target arrival position is [-0.4, 0.13, 0.46], measured in meters. The motion trajectory is a straight line connecting the initial position to the target position. To ensure that the end-effector moves along the designated path, 383 waypoints were inserted along the motion trajectory. Our method provides inverse kinematics (IK) solutions at a frequency of 10Hz, while the Franka Panda robotic arm receives control signals at a frequency of 1,000 Hz. To accommodate this, we performed B-spline linear interpolation along the trajectory, resulting in a total of 38,500 points in the final path, including the starting and ending points.

**Figure 6:**
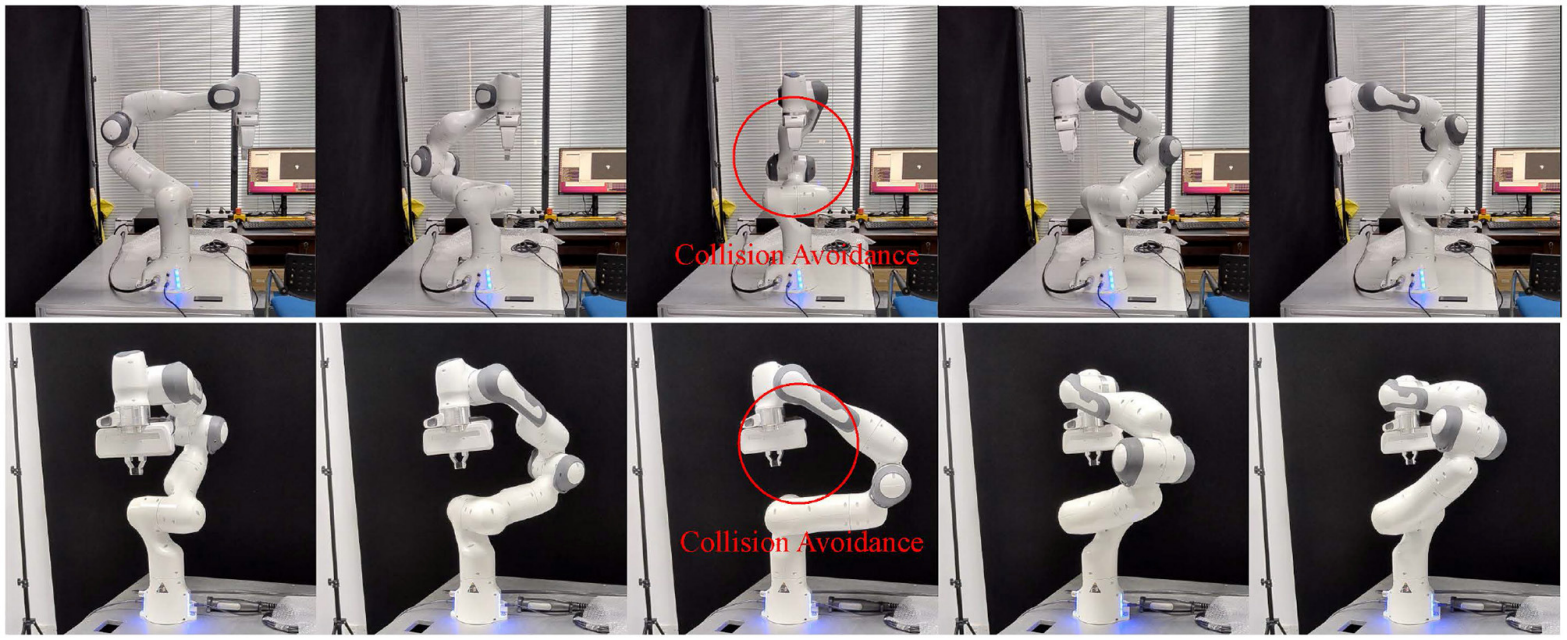
The upper row is the right view of the motion process, the lower row is the front view of the motion process.

Figure 6 shows the motion process of the robotic arm from two views. Under the guidance of our controller, the robotic arm adjusts the linkage position and bypasses the end-effector to avoid collisions.

## 6 Conclusion

Introducing reinforcement learning into the field of robot control remains challenging. This encompasses searching for suitable solutions within complex solution spaces and ensuring smooth robot motions. This paper proposes a reinforcement learning-enhanced pseudo-inverse method for robotic arm control, aiming to maintain the smoothness of robot motions with self-collisions avoided. Although our current work is focuses solely on the inverse kinematics of the end effector position, there is potential for broader applications in the context of redundant robotic arms, such as in human-robot collaboration or medical scenarios.

## Data availability statement

The raw data supporting the conclusions of this article will be made available by the authors, without undue reservation.

## Author contributions

TH: Writing – original draft, Writing – review & editing. WL: Writing – review & editing. KH: Writing – review & editing.
